# The Ethics of Facial Allotransplantation: A Systematic Review

**DOI:** 10.1097/GOX.0000000000002425

**Published:** 2019-10-31

**Authors:** Xiangxia Liu, Sarah Langsdon, Wesley Holloway, Shuqia Xu, Qing Tang, Yangbin Xu, Sai Ram Velamuri, William Hickerson

**Affiliations:** From the * Division of Plastic Surgery, First Affiliated Hospital, Sun Yat-sen University, Guangzhou, China; † Department of Plastic Surgery, University of Tennessee Health Science Center, Memphis, Tenn.; ‡ College of Medicine, University of Tennessee Health Science Center, Memphis, Tenn.; § Health Sciences Library, University of Tennessee Health Science Center, Memphis, Tenn.

## Abstract

Currently, there are more than 40 cases of facial allotransplantation performed by 13 different groups in 10 countries. Although it has become a potential option to reconstruct and restore the function and appearance of severely facially disfigured individuals, the ethical concerns of facial allotransplantation remain unsolved. We conducted a systematic review to better understand the ethical concerns on facial allotransplantation and the changing trends of the ethical debate over time.

**Methods:**

A systematic review of 3 databases was performed to identify articles related to ethical topics on facial allotransplantation. The inclusion criteria were peer-reviewed articles written since 1995 on the topics of ethics and facial allotransplantation in English, French, and Chinese languages. The ethical concerns extracted from the included articles were categorized into 4 core principles of ethics: autonomy, beneficence, nonmaleficence, and justice. The different themes under these 4 principles were extracted and subgrouped. The positions of the included articles were collected. Joinpoint regression was applied to compare the frequency of themes and positions by publication year. We presented the main topics on ethical concerns and the changing trends in ethical themes and principles of facial allotransplantation.

**Results:**

There were 889 articles identified initially. After excluding 265 duplicated articles, 624 articles were included for title/abstract review process, and 148 articles were included in final data analysis. The publication year was from 2002 to 2018 with 136 articles in English, 11 in French, and 1 in Chinese. The most addressed principle was nonmaleficence (117/148, 79.1%), followed by beneficence (116/148, 78.4%), justice (103/148, 69.6%), and autonomy (86/148, 58.1%). The themes on immunosuppression/rejection, quality of life, and identity were the top 3 addressed ethical concerns. Twelve of 13 most addressed ethical themes demonstrated a decreasing trend after 2004. The themes of identity under beneficence showed a significant decrease after 2004. Ethical concerns on the cost/financial topic were the only one showing consistently increase trends from 2002 to 2018. There was a significant increase of the papers in favor of facial allotransplantation procedure comparing to those were against or neutral before and after 2008.

**Conclusions:**

More and more articles support facial allotransplantation as a feasible option to reconstruct and restore the function and appearance of severely facially disfigured individuals. The requirement of life-long immunosuppression therapy, quality of life, and identity center the ethical debates. Supported by favorable short-term results, 12 of 13 most addressed ethical concerns have trended down. The theme of cost/financial topic becomes more frequently addressed in recent years.

## Abstract 2

Supplemental Digital Content is available in the text.

## INTRODUCTION

The human face is a complex 3-dimensional structure that is central to human identity.^1,2^ Facial appearance identifies our gender, age, and ethnicity; it conveys our emotions and allows us to interact with our surrounding world.^3^ When the essential features and functions of the human face are damaged, it has not only physical but emotional and psychological consequences. With advancing technological and available immunosuppressive regimens, facial allotransplantation for severely disfigured patient became possible. The ethics on facial allotransplantation has been at the forefront of the ongoing debate even before the world’s first successful case in France in 2005. Within the past 13 years, the field has expanded remarkably. More than 40 cases were reported from 10 different countries, including France, China, United States, Spain, Belgium, Turkey, Poland, Russia, Finland, and Canada.^4–6^

In 2016, Isabelle Dinoire, the world’s first face transplant patient, died after a long illness, adding one more to a total 7 deaths so far.^7^ Like her initial introduction into the spotlight, her death perpetuated the ongoing debate. In early 2018, a French team performed the second facial allotransplantation on a patient who lost his graft due to chronic rejection.^8^ These newly monumental developments in facial allotransplantation add more valuable data to the ethical debate and could shift the trends potentially. In this article, we performed a systematic review of the ethics on facial allotransplantation, collected the data of 4 core principles of bioethics: autonomy, beneficence, nonmaleficence, and justice, the authors’ positions on facial allotransplantation, and presented the changing trends in ethical themes, principles, and positions of facial allotransplantation over time.

## MATERIALS AND METHODS

We conducted a literature search in 3 databases (PubMed, Scopus, and Cochrane) from 1995 to October 23, 2018. The literature was searched with specific search strategies designed following the systematic review guidelines and with the assistance of the health sciences librarian (W.H.). The search strings concerned the concepts of the face, allotransplantation, and ethics for the topic of the systematic review. The search strings were constructed by combining controlled vocabulary and keyword terms (see pdf, Supplemental Digital Content 1, which displays the search strategy used, http://links.lww.com/PRSGO/B224). Inclusion criteria were peer-reviewed articles on face allotransplantation and the ethical topics relating to from 1995 to the present, and languages were limited to English, French, or Chinese. Two reviewers (X.L., S.L.) performed the title/abstract screening and review of full-text articles. Any disagreements regarding articles to be excluded and included were resolved by discussion. Ethical themes were extracted qualitatively and categorized under 4 principles of bioethics: autonomy (allows the patient to “self-rule,” free from interference by others), beneficence (the moral obligation to benefit others), nonmaleficence (the obligation to not cause harm to others), justice (provides fair and appropriate treatment to all persons regardless of status or special considerations).^9^ The positions of the articles were defined as “yes” or “no” by looking for the specific statement regarding facial allotransplantation in the articles; if there was no such statement, it was defined as “neutral.”

The frequency of ethical themes/principles and the position of the articles were assessed. The Joinpoint regression program (Version 4.6.0.0, National Cancer Institute, Calverton, Md.) was applied to analyze the trends and its annual percentage change (APC) in most common ethical themes and position of the articles. The χ^2^ test was used to determine whether there was a statistically significant difference between 3 category groups of the position. *P* value <0.05 was set as the level of significance.

## RESULTS

**Fig. 1.**
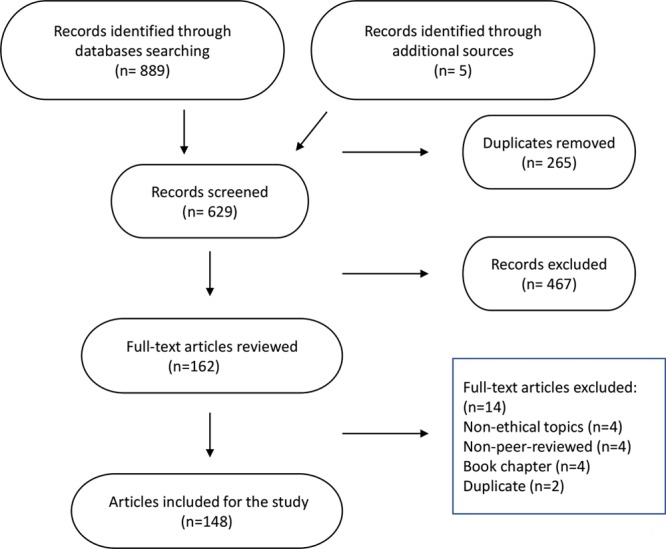
Flow chart shows the results of screening process and final article inclusion.

The initial search discovered 889 citations. After removing 265 duplicates, 624 citations were included in the title/abstract review, 467 articles were excluded during this process, and 157 articles were included for full-text review. There were 5 additional articles included into the full-text review by manual searching of the references. Following the full-text review, 148 articles were included in final data analysis (Fig. 1).

**Fig. 2.**
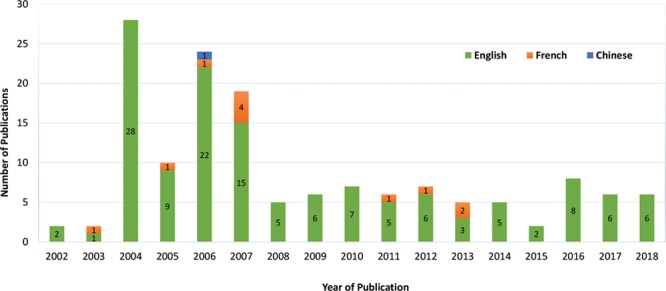
Number of articles (n = 148) addressing the ethical principle on facial allotransplantation.

**Fig. 3.**
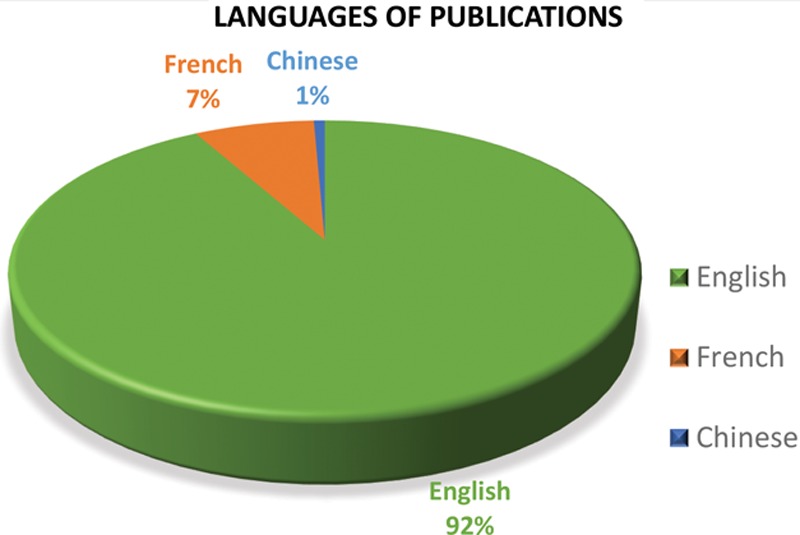
Percentage of the published articles based on language.

The first publication on the ethics of facial allotransplantation was in 2002.^10^ The least number of publications per year were in 2002, 2003, and 2015 with only 2 papers, the most number of publications per year was in 2004 with 28 papers, followed by 2006 with 22 papers. The average number of publications per year was 9 (Fig. 2). There were 136 English, 11 French, and 1 Chinese publications (Fig. 3). In all 148 articles included, nonmaleficence was the most frequent ethical principle addressed (117/148, 79.1%),^1–3,7,10–121^ followed by beneficence (116/148, 78.3%),^1–3,5,7,10,13–24,26–29,32–42,44,46,48,49,51–56,58,60,61,63–66,69–84,86,87,89–93,95–137^ justice (103/148, 69.6%),^2,3,5,7,11,14,17–22,24–28,30–32,34,35,38,39,41–44,46,48,49,51,52,54,55,57,58,60–64,66–73,75–77,79,82–85,87–92,94,95,97–102,104,105,107–112,114,117,119–121,123,125,129–131,135,138–147^ and autonomy (86/148, 58.1%).^1–3,5,7,12,14–17,19,23–25,27,28,30,32,34,35,37–41,44,46,48,49,53,56,58,61–66,69–74,79,80,82–84,88,91,94,95,97,98,100–105,107,109,111,113,116,117,119–121,124,125,130,131,133,136,138,140–143,145,147–149^

**Fig. 4.**
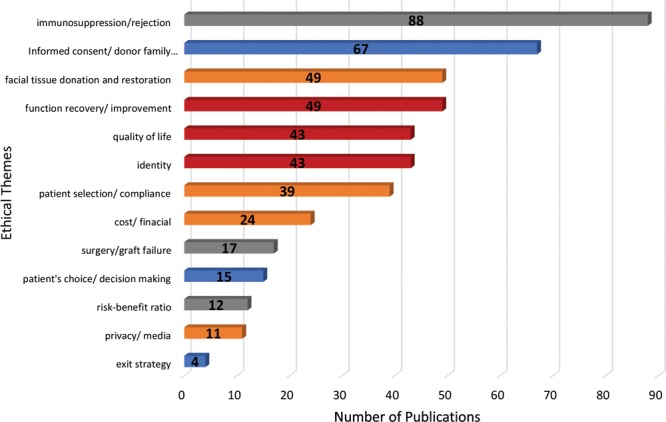
Number of articles addressing the ethical themes.

**Fig. 5.**
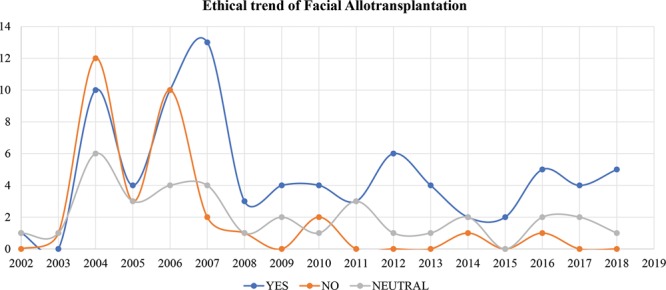
The annual percentage change trends of the position of the included articles from 2002 to 2018.

The most frequent addressed themes under the principles were risk of immunosuppression or rejection (n = 88, 59.5%),^1–3,7,10–12,14,16–19,22,23,25,29–41,43–56,58–64,66,67,69,71,72,75–79,81,83–85,87–92,95,97,100,102–106,108–112,114,115,117–119,121^ followed by informed consent or donor family consent (n = 67,45.3%),^1–3,5,12,14,15,23,25,27,28,32,34,35,38–41,44,46,48,49,53,56,58,61,63–66,69–74,80,82–84,88,94,97,100–105,107,111,116,117,120,121,130,131,133,136,138,141–143,145,147,149^ facial tissue donation and restoration (n = 49, 32.9%),^2,11,14,18,21,22,25,28,30,34,39,41–43,46,48,49,51,55,60,64,66–69,71,75,76,83,84,86,89,97,98,100,105,107,108,110,111,119,123,125,131,135,140,143–145^ functional recovery or improvement (n = 49,33.1%),^2,5,10,17–19,29,32,37,38,42,48,53,55,58,61,63,64,66,69–71,75,76,82,84,86,87,90,92,95,96,98,102,104,106–108,110,111,117,121,129–133,135,137^ quality of life (n = 43, 29.1%),^3,14,26–29,37,39–41,44,46,49,54,55,66,72,73,77,78,83,86,93,96–99,101,103,104,106,108,111,113,114,117–119,128,133–136^ and identity (n = 43, 28.1%)^1,7,13,14,19,22,23,33,37,41,42,56,60,61,66,70,72,73,76,78,80,83,84,87,101,105,107,111,112,115,118,122,123,126–128,131,132,135–137^ (Fig. 4). The APC trends calculated for the 13 most frequent addressed themes showed 11 themes decreased in frequency overtime (APC < −1), 4 themes increased (APC > 1), and 1 theme remained the same (−1< APC < 1). Three themes including informed consent, identity, and quality of life presented APC trend changes from increase to decrease before and after the year of 2004, and only the theme of identity demonstrated a significant decrease between 2004 and 2018 (APC = −11.1, *P* < 0.05) (Fig. 5). The theme of cost was the only one that showed a consistent increase from 2002 to 2018 (APC = 6.0) (Table 1).

**Table 1.** Joinpoint Analysis of Number of Papers Addressing Ethical Themes from 2002 to 2018

Principle	Theme	Start of Trend	End of Trend	APC	95% CI	*P*
Autonomy	Informed consent/donor family consent	2002	2004	296.4	−49.5 to 3,012.9	NS
		2004	2018	−8.3	−16.8 to 1.0	NS
	Patient’s choice/decision making	2002	2018	−1.8	−9.5 to 6.5	NS
	Exit strategy	2002	2018	−2.8	−6.6 to 1.1	NS
Beneficence	Identity	2002	2004	228.9	−63.2 to 2,838.0	NS
		2004	2018	−11.1	−19.8 to −1.5	<0.05*
	Quality of life	2002	2004	235.1	−39.4 to 1,753.7	NS
		2004	2018	−6.1	−13.3 to 1.8	NS
	Functional recovery/improvement	2002	2018	−1.6	−9.8 to 7.4	NS
Nonmaleficence	Immunosuppression/rejection	2002	2018	−3.4	−11.5 to 5.4	NS
	Risk-benefit ratio	2002	2018	−5.3	−11.1 to 0.9	NS
	Surgery/graft failure	2002	2018	−2.3	−9.8 to 5.7	NS
Justice	Cost/financial	2002	2018	6.0	−1.1 to 13.6	NS
	Patient selection/compliance	2002	2018	0.3	−8.4 to 9.8	NS
	Facial tissue donation and restoration	2002	2018	−5.5	−14.4 to 4.3	NS
	Privacy/media	2002	2018	−2.8	−8.2 to 2.9	NS

CI, confidence interval; NS, not significant.

A total of 54.1% of the publications tended to support facial allotransplantation (n = 80), 22.3% of publications tended to oppose it (n = 33), and 23.6% showed neutral position (n = 35). As shown in Figure 5, there was a turning point at the year of 2008. Chi-squared test results demonstrated that there was statistically significant difference when comparing “yes” group to both “no” group and “ neutral” group before and after the year of 2008 (*P* < 0.05), whereas there was no significant difference between “no” group and “neutral” group before and after year of 2008.

## DISCUSSION

The ethics debate on facial allotransplantation started in the early 2000s when the first article on ethics of facial allotransplantation was published.^10^ Initially, both the Royal College of Surgeons of England and the National Consultative Ethics Committee for Health and Life Science from France stated that the timing for facial allotransplantation was not suitable at that moment due to the unsolved concerns about how to obtain fully valid informed consent, the risk of life-long immunosuppressive therapy, and the potential psychologic impact on the recipient.^14,27^ In 2004, the Louisville team published the ethical guidelines for facial allotransplantation and claimed they are ready for the procedure.^18^ At the same year, the Cleveland Clinic team received the world first institutional review board approval to perform human facial allotransplantation. These sparked fierce debates on the ethics topic of facial allotransplantation in medical community.^2,15–17,19–22,24,25,27–29,31–35,122,123,138,148,150^ As shown in our search results, there were 28 articles published in 2004 representing the highest output year from 2002 to 2018. With limited data and research on the procedure, most of the parties were either against or neutral to facial allotransplantation. Only 34.3% (11/32) of the parties were in favor to the procedure before 2005. The ethical debates did not trend down after the first successful human facial allotransplantation performed in France in 2005,^151^ instead the transplant brought up another round of debate not only within the medical community this time but also the general populations and the media.^44,48–50^ As shown in the study, 24 papers were published in 2006 representing the second highest number on a yearly basis.^46,56,59,128^ Although this time the debate involved some discussion of surgical techniques, the ethical topics still centered the stage. The England team reiterated that “until there is further research and the prospect of better control of the complications, it would be unwise to proceed with human facial transplantation.”^49^ The French team stated that “a full facial CTA does not make sense at present.”^48^ The United States team encouraged “further research work in improving transplant immunology and analyzing the long-term results.”^152^

Despite the unfavorable opinions from both profession societies and the public, 3 different research groups from China, United States, and France successfully carried out another 3 cases of facial allotransplantation from 2006 to 2008.^153–155^ The APC change results showed there was a turning point in year 2008 regarding the positions of the included articles (Fig. 5). Only 45.6% (41/90) of the articles were in favor of the procedure before 2008, whereas >67.2% (39/58) supported it after 2008. There was a statistically significant increase of the articles in favor of facial allotransplantation before and after 2008 comparing to other 2 groups (*P* < 0.05). In the next 4 years, from 2009 to 2012, nearly 2 dozen cases were performed worldwide and supported by the favorable short-term result. Facial allotransplantation has become a potential treatment option for carefully selected patients.^152^ This shift was shown clearly in our study that there were only 2 articles against facial allotransplantation procedures after 2010 (Fig. 5). In the first article, Flynn et al.^101^ stated that technological readiness was insufficient for the implementation of facial transplantation within pediatrics. The second article addressed social anonymity which was almost impossible for facial allotransplantation cases.^146^

Encouraged by the favorable functional and aesthetic outcome, more and more groups joined the research and the number of facial allotransplantation climbed to 38 cases worldwide from 2010 to 2016. Although huge advancements were achieved in the research of facial allotransplantation, the ethical debate continued. On April 22, 2016, Isabelle Dinoire, the world’s first facial allotransplantation recipient, died after a long illness, adding one more to a total 7 deaths so far.^7^ On January 2018, a French team performed the second facial allotransplantation on a patient who lost his graft due to chronic rejection.^8^ During our preparation of the article, the Italian team encountered acute rejection and total graft loss in the country’s first facial allotransplantation case.^156^ All these recent incidences reminded us this type of procedure is still in its infancy and associated with significant morbidity and mortality. Patients are at risks of graft rejection, immunosuppressive therapy–associated complications even years after the initial operation.

**Fig. 6.**
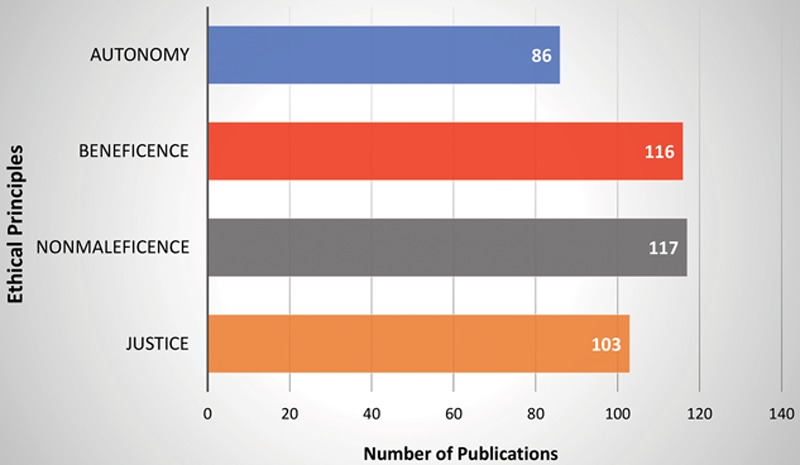
Number of articles addressing the principles of ethics.

Based on our study, the nonmaleficence (the obligation to not cause harm to others) was the most addressed ethical principle, appearing in 79.1% (117/148) of the articles, followed by 78.4% (116/148) in beneficence (the moral obligation to benefit others), 69.6% (103/148) in justice (provides fair and appropriate treatment to all persons regardless of status or special considerations), and 58.1% in autonomy (allows the patient to “self-rule,” free from interference by others) (Fig. 6). These results reflected the natural characteristic of the life-enhancing procedure compared with other life-saving solid organ transplantation procedures. Similar results were found in the research of hand allotransplantation.^157^ Among the 13 most addressed themes, immunosuppression/rejection (n = 88), informed consent/donor family consent (n = 67), facial tissue donation and restoration (n = 49), functional recovery/improvement (n = 49), quality of life (n = 43), and identity (n = 43) were among the leading concerns (see details in Fig. 4). From 2002 to 2004, the APC trends of the informed consent/donor family consent (APC = 296.4), identity (APC = 228.9), and quality of life (APC = 235.1) were far beyond +1 which indicated these 3 themes were the most increasingly discussed ethical concerns at that time. After 2004, all the themes except the cost/financial under justice showed a decreased trend. These findings could be interpreted that the ethical concerns on facial allotransplantation procedure were partially relieved as more and more cases were done and with a favorable functional and aesthetic outcome. Among them, the theme of identity under beneficence showed significant decrease in APC trend (*P* < 0.05), indicated the identity had become a less and less concerned ethical topic (Table 1). This finding was further supported by the fact that all facial allotransplantation recipients reported adapted to their new identity without difficulty. The theme of cost/financial under justice was the only one had consistently increase in trend (APC = 6.0) from 2002 to 2018. This indicated that the theme of cost/financial had become a more and more discussed ethical topic. Toure et al.^60^ estimated the total cost of facial allotransplantation procedure to be between $250,000 and $1,500,500 in the French system. Siemionow et al.^158^ demonstrated that the cost of conventional reconstructive procedures and the cost of facial allotransplantation procedure in the first US case was similar between $250,000 and $350,000. This amount did not include the cost of life-long immunosuppression which was estimated at $20,000 per year.^110^ Currently, facial allotransplantation cases were supported by either research funding or an institutional budget and each case was funded on an individual basis. Fortunately, as discussed at the 6th Biennial American Society for Reconstructive Transplantation meeting in Chicago on November 2018, the researchers were planning for the application of new Current Procedural Terminology code for facial allotransplantation procedure and other vascularized composite tissue allotransplantation procedures which were the first step to get possible insurance coverage. The researchers agreed that it may be unrealistic to have commercial insurance coverage before facial allotransplantation procedures could become a standard option for the reconstruction of severely disfigured patients. Considering the high cost and the patient’s mental and physical suffering from numerous conventional reconstructive procedures, it would be possible to have Medicare/Medicaid coverage on a case by case basis in the near future.

Often, information is only available through the public media with no official data from the surgical teams.^159^ For example, some of the facial allotransplantation cases done by the teams from Turkey and the case done by the Russian team were not reported in the medical literature. One of the limitations of our study is, although we included English, French, and Chinese, we were still missing Spanish, Turkish, Polish, Russian, and Finnish language, in which its case population consisted of at least one-third of the total case volume. This issue may become more apparent when there is significant under-reporting of a large number of cases in the medical literature, especially in recent years. Another limitation is the difference number of library search during the long process of preparation of the article. To reduce this bias, we performed a follow-up search 6 months after our initial search on April 2018. Not surprising, we yielded 5 more articles into the final data analysis.

## CONCLUSIONS

Although facial allotransplantation has been proved to be a potential option to reconstruct and restore the function and appearance of patients with devastating facial injuries, the unsolved ethical debates on this life-enhancing procedure continue. Supported by favorable short-term outcomes, the ethical concerns on immunosuppression/rejection, quality of life, and identity tended to decrease sharply, especially on the theme of identity after 2004. To better address the increasing concerns on the cost and financial-related topic, the researchers should work together to strive for the coverage from governmental insurances.

## ACKNOWLEDGMENT

The authors appreciate Dr. Ren Dongren for his valuable help during the statistical analysis of the data.

## Supplementary Material
