# Supplementary material for: The Ethics of Facial Allotransplantation: A Systematic Review
Source: Plast Reconstr Surg Glob Open. 2019 Oct 31;7(10):e2425. doi: 10.1097/GOX.0000000000002425 (PMC6846319; doi:10.1097/GOX.0000000000002425)
Supplement: Supplementary file 1 [file gox-7-e2425-s001.pdf]

The ethics on facial allotransplantation: a systematic review

Supplement

Search Strategy

PubMed

((face [Mesh]) OR (face) OR (faces) OR (facial) OR (ear [Mesh]) OR (ear) OR (ears) OR (scalp [Mesh]) OR (scalp) OR (scalps)) ((vascularized composite allotransplantation [Mesh:NoExp]) OR (vascularized composite allotransplant*) OR (VCA) OR (facial transplantation [Mesh]) OR (facial transplant*) OR (allotransplant*) OR (composite tissue allotransplantation) OR (composite tissue allotransplant*) OR (transplantation) OR (transplants [Mesh:NoExp]) OR (transplant*) OR (organ transplantation [Mesh]) OR (organ transplant*) OR (organ transplantation) OR (tissue transplantation [Mesh:NoExp]) OR (tissue transplant*)) ((ethics [Mesh:NoExp]) OR (ethic*) OR (ethical issues) OR (ethical concern*) OR (morals [Mesh:NoExp]) OR (morals) OR (moral*) OR (virtues [Mesh]) OR (virtues) OR (virtue*) OR (principle-based ethics [Mesh]) OR (principle based ethics) OR (ethics, professional [Mesh:NoExp]) OR (professional ethic*) OR (ethics, clinical [Mesh:NoExp]) OR (clinical ethic*) OR (ethics, medical [Mesh:NoExp]) OR (medical ethic*) OR (personhood [Mesh]) OR (bioethical issue*) OR (bioethical concern*) OR (bioethics [Mesh:NoExp]) OR (bioethic*) OR (nonmaleficen*) OR (autonomy) OR (personal autonomy) OR (social justice) OR (justice) OR (beneficen*) OR (dignity))

Searched: April 26, 2018
Citations retrieved: 377

Follow up search: Oct 23,2018
Citations retrieved: 379

Search String Details

Terms	Search Strings	Results	Date
Face	((face [Mesh]) OR (face) OR (faces) OR (facial) OR (ear [Mesh]) OR (ear) OR (ears) OR (scalp [Mesh]) OR (scalp) OR (scalps))	622,751	4/26/2018
Allotransplantation	((vascularized composite allotransplantation [Mesh:NoExp]) OR (vascularized composite allotransplant*) OR (VCA) OR (facial transplantation [Mesh]) OR (facial transplant*) OR (allotransplant*) OR (composite tissue allotransplantation) OR	803,744	4/26/2018

	(composite tissue allotransplant*) OR (transplantation) OR (transplants [Mesh:NoExp]) OR (transplant*) OR (organ transplantation [Mesh]) OR (organ transplant*) OR (organ transplantation) OR (tissue transplantation [Mesh:NoExp]) OR (tissue transplant*))		
Ethics	((ethics [Mesh:NoExp]) OR (ethic*) OR (ethical issues) OR (ethical concern*) OR (morals [Mesh:NoExp]) OR (morals) OR (moral*) OR (virtues [Mesh]) OR (virtues) OR (virtue*) OR (principle-based ethics [Mesh]) OR (principle based ethics) OR (ethics, professional [Mesh:NoExp]) OR (professional ethic*) OR (ethics, clinical [Mesh:NoExp]) OR (clinical ethic*) OR (ethics, medical [Mesh:NoExp]) OR (medical ethic*) OR (personhood [Mesh]) OR (bioethical issue*) OR (bioethical concern*) OR (bioethics [Mesh:NoExp]) OR (bioethic*) OR (nonmaleficen*) OR (autonomy) OR (personal autonomy) OR (social justice) OR (justice) OR (beneficen*) OR (dignity))	342,988	4/26/2018
((#2 AND #3) AND #4)		377	4/26/2018
Follow-Up Search	((#2 AND #3) AND #4)	379	10/23/2018

Scopus

TITLE-ABS-KEY ((face) OR (faces) OR (facial)) AND TITLE-ABS-KEY ((vascularized AND composite AND allotransplant*) OR (vca) OR (allotransplant*) OR (transplant*) OR (composite AND tissue AND allotransplant*) OR (cta) OR (composite AND tissue AND transplant*) OR (organ AND transplant*) OR (tissue AND transplant*)) AND TITLE-ABS-KEY ((ethic*) OR (ethic* AND issue*) OR (ethic* AND concern*) OR (principle AND based AND ethics) OR (moral*) OR (virtue*) OR (personhood) OR (social AND justice) OR (justice) OR (nonmaleficen*) OR (personal AND autonomy) OR (autonomy) OR (beneficen*) OR (dignity) OR (bioethic*) OR (bioethic* AND issue*) OR (bioethic* AND concern*) OR (profession* AND ethic*) OR (medical AND ethic*) OR (clinical AND ethic*))

Searched: April 26, 2018
Citations retrieved: 480

Follow up search: Oct 23,2018
Citations retrieved: 502

Search String Details

Terms	Search Strings	Results	Date
Face	TITLE-ABS-KEY ((face) OR (faces) OR (facial))	801,277	4/26/2018
Allotransplantation	TITLE-ABS-KEY ((vascularized AND composite AND allotransplant*) OR (VCA) OR (allotransplant*) OR (transplant*) OR (composite AND tissue AND allotransplant*) OR (CTA) OR (composite AND tissue AND transplant*) OR (organ AND transplant*) OR (tissue AND transplant*))	848,678	4/26/2018
Ethics	TITLE-ABS-KEY ((ethic*) OR (ethic* AND issue*) OR (ethic* AND concern*) OR (principle AND based AND ethics) OR (moral*) OR (virtue*) OR (personhood) OR (social AND justice) OR (justice) OR (nonmaleficen*) OR (personal AND autonomy) OR (autonomy) OR (beneficen*) OR (dignity) OR (bioethic*) OR (bioethic* AND issue*) OR (bioethic* AND concern*) OR (profession* AND ethic*) OR (medical AND ethic*) OR (clinical AND ethic*))	713,186	4/26/2018

((#2 AND #4) AND #5)		480	4/26/2018
Follow-Up	((#2 AND #4) AND #5)	502	10/23/2018

Cochrane Library

Searched: April 26, 2018
Citations retrieved: 0

Follow up search: Oct 23,2018
Citations retrieved: 0

Search String Details

**Terms**	**Search Strings**	**Results**	**Date**	**Search Lines in Cochrane**
**Allotransplantation/ Transplantation, Facial**	MeSH descriptor: [Facial Transplantation] explode all trees	2	4/26/2018	#1
**Face**	MeSH descriptor: [Face] explode all trees	3,183	4/26/2018	#2
**Ear**	MeSH descriptor: [Ear] explode all trees	1,154	4/26/2018	#3
**Scalp**	MeSH descriptor: [Scalp] explode all trees	289	4/26/2018	#4
**Face/Ear/Scalp with Keywords**	((#2 or #3 or #4 or face or faces or facial or ear or ears or scalp or scalps) near/3 (transplant* or vascularized composite allotransplant* or VCA or allotransplant* or composite tissue allotransplant* or organ transplant* or tissue transplant*))	54	4/26/2018	#5
	(#1 or #5)	54	4/26/2018	#6
**Ethics**	MeSH descriptor: [Ethics] explode all trees	619	4/26/2018	#7
**Morals**	MeSH descriptor: [Morals] explode all trees	786	4/26/2018	#8
**Virtues**	MeSH descriptor: [Virtues] explode all trees	1	4/26/2018	#9

**Ethics/Morals/ Virtues with Keywords**	(#7 or #8 or #9 or ethic* or moral* or bioethic* or bioethic* issue* or bioethic* concern* or personhood or clinical ethic* or medical ethic* or professional ethic* or principle-based ethic* or principle based ethic* or beneficen* or nonmaleficen* or autonomy or personal autonomy or social justice or justice or virtue* or dignity)	15,689	4/26/2018	#10
	**(#6 and #10)**	**0**	4/26/2018	
Follow-Up	(#6 and #10)	**0**	10/23/2018	
